# Protocol for the Prognostication of Consciousness Recovery Following a Brain Injury

**DOI:** 10.3389/fnhum.2020.582125

**Published:** 2020-11-12

**Authors:** Catherine Duclos, Loretta Norton, Geoffrey Laforge, Allison Frantz, Charlotte Maschke, Mohamed Badawy, Justin Letourneau, Marat Slessarev, Teneille Gofton, Derek Debicki, Adrian M. Owen, Stefanie Blain-Moraes

**Affiliations:** ^1^School of Physical and Occupational Therapy, McGill University, Montreal, QC, Canada; ^2^Montreal General Hospital, McGill University Health Centre, Montreal, QC, Canada; ^3^Department of Psychology, King’s University College at Western University, London, ON, Canada; ^4^The Brain and Mind Institute, Western University, London, ON, Canada; ^5^Department of Psychology, Western University, London, ON, Canada; ^6^Integrated Program in Neuroscience, McGill University, Montreal, QC, Canada; ^7^Department of Anesthesia, McGill University, Montreal, QC, Canada; ^8^Department of Neurology and Neurosurgery, Division of Neurocritical Care, McGill University Health Center, Montreal, QC, Canada; ^9^Montreal Neurological Hospital, McGill University Health Center, Montreal, QC, Canada; ^10^Department of Medicine, Schulich School of Medicine and Dentistry, Western University, London, ON, Canada; ^11^Department of Clinical Neurological Sciences, Schulich School of Medicine and Dentistry, Western University, London, ON, Canada; ^12^Neurocritical Care Program, Division of Neurology, Department of Clinical Neurological Sciences, Western University, London, ON, Canada; ^13^Department of Physiology and Pharmacology, Western University, London, ON, Canada

**Keywords:** intensive care unit, EEG, brain injury, continuous sedation, coma, cognitive testing, consciousness, prognosis

## Abstract

Individuals who have suffered a severe brain injury typically require extensive hospitalization in intensive care units (ICUs), where critical treatment decisions are made to maximize their likelihood of recovering consciousness and cognitive function. These treatment decisions can be difficult when the neurological assessment of the patient is limited by unreliable behavioral responses. Reliable objective and quantifiable markers are lacking and there is both (1) a poor understanding of the mechanisms underlying the brain’s ability to reconstitute consciousness and cognition after an injury and (2) the absence of a reliable and clinically feasible method of tracking cognitive recovery in ICU survivors. Our goal is to develop and validate a clinically relevant EEG paradigm that can inform the prognosis of unresponsive, brain-injured patients in the ICU. This protocol describes a study to develop a point-of-care system intended to accurately predict outcomes of unresponsive, brain-injured patients in the ICU. We will recruit 200 continuously-sedated brain-injured patients across five ICUs. Between 24 h and 7 days post-ICU admission, high-density EEG will be recorded from behaviorally unresponsive patients before, during and after a brief cessation of pharmacological sedation. Once patients have reached the waking stage, they will be asked to complete an abridged Cambridge Brain Sciences battery, a web-based series of neurocognitive tests. The test series will be repeated every day during acute admission (ICU, ward), or as often as possible given the constraints of ICU and ward care. Following discharge, patients will continue to complete the same test series on weekly, and then monthly basis, for up to 12 months following injury. Functional outcomes will also be assessed up to 12 months post-injury. We anticipate our findings will lead to an increased ability to identify patients, as soon as possible after their brain injury, who are most likely to survive, and to make accurate predictions about their long-term cognitive and functional outcome. In addition to providing critically needed support for clinical decision-making, this study has the potential to transform our understanding of key functional EEG networks associated with consciousness and cognition.

## Introduction

Individuals who have suffered a severe brain injury typically require extensive hospitalization in intensive care units (ICUs) to survive their injury and regain their cognitive functions. In the acute stage post-injury, the decisions made by the ICU healthcare teams have an enormous impact on patient survival and outcomes. Importantly, decisions regarding the treatment course and goals of care are often based on behavioral responses, which are highly dependent on a multitude of clinical and environmental factors, rather than on objective and quantifiable markers. There is thus a critical need to develop systems and techniques that can be deployed at the bedside to predict patient outcome, and thus inform clinical decision-making about treatments of unresponsive brain-injured patients in the ICU.

There are two major barriers to developing and deploying a point-of-care system for predicting outcomes of brain-injured patients. First, there is currently no accepted set of robust prognostic markers that can be gathered at the bedside when patients are unresponsive. Though fMRI is an attractive option, most patients are too vulnerable within the first days post-admission to be moved to the fMRI scanner (Weijer et al., 2016). While the brain responses of these patients are routinely monitored through electroencephalography (EEG) and visually inspected by a trained neurologist to identify pathological characteristics, the EEG waveforms and spectral properties have limited prognostic value with respect to cognitive outcomes, beyond predicting patient survival. Second, very little is known about the long-term outcomes of ICU survivors. While it is known that long-term cognitive impairments affect 40–100% of ICU survivors (Hopkins et al., 2005; Moulaert et al., 2009; Iwashyna et al., 2010; Wilcox et al., 2013; Honarmand et al., 2020), and affects people of all ages (Pandharipande et al., 2014), there is a lack of a systematic, patient-accessible method for accurately tracking cognitive recovery in these patients. Our group has recently established that a web-based cognitive battery that can be self-administered by patients is both feasible for use in ICU patients and accurately detects cognitive impairment across multiple cognitive domains (Honarmand et al., 2019). In the absence of this cognitive recovery data, it is impossible to characterize the prognostic value of any markers gathered in the ICU from this population.

In current clinical practice, predictions of outcome in brain-injured patients vary based on the etiology of coma. Demographic data, history, brainstem examination, behavioral measures, and motor responses (e.g., Glasgow Coma Scale) (Teasdale and Jennett, 1974), electrophysiology (EEG, somatosensory evoked potentials), and neuroimaging findings are all considered. Though these measures are accessible, some have a contested prognostic value, especially within the first days post-injury (Kouloulas et al., 2013). Brain responses gathered through EEG have shown some promise in predicting outcomes in this population. In particular, the presence of the mismatch negativity (MMN) event-related brain potential (ERP) has been associated with awakening from a state of coma (Fischer et al., 1999; Morlet and Fischer, 2014). While the presence vs. absence of this brain response has high specificity (>90%), it has an extremely low sensitivity (<30%), and thus has limited value in clinical practice. Other long-latency ERPs such as the P300 and N400 have also been tested for prognostic purposes but have shown poor sensitivity and specificity (Wang et al., 2011).

Preliminary studies from our team have suggested the potential prognostic value of network features of continuous EEG and the reorganization of these features upon a change of sedation/anesthesia status (Blain-Moraes et al., 2017; Nadin et al., 2020). Instead of focusing on waveforms and spectral properties (as measured in clinical EEG) or on event-based responses, information flow networks in the brain (e.g., functional connectivity and graph properties) and their changes upon administration or removal of anesthesia have heralded the return (or not) of patient consciousness. These network-based features of EEG have also shown positive predictive values of an unresponsive individual’s level of consciousness in patients exposed to anesthesia, in other recent studies (Boly et al., 2011; Lee H. et al., 2013; Lee U. et al., 2013; Chennu et al., 2016). While promising, the full prognostic potential of these features have yet to be explored.

The use of EEG network features as a prognostic tool must be validated against a set of patient outcome measures that are sensitive to the dynamic and complex changes in cognitive functions across multiple domains in ICU survivors. Such measures have historically been difficult to gather due to a lack of comprehensive, easy-to-administer neurocognitive tests. Currently, assessment of cognitive function requires that patients attend a clinic where specially trained personnel administer standard cognitive batteries. This model has several limitations including the length of these testing sessions, patient inconvenience of traveling to clinic assessments, high costs associated with employing trained personnel, and high rates of patient attrition. As a result, traditional methods of comprehensive cognitive assessment cannot be used for a large-scale multi-center natural history study that requires repeated measurement of cognitive function within individual patients during their recovery (Honarmand et al., 2020).

Over the last 25 years, a suite of computerized cognitive tests, named Cambridge Brain Sciences (CBS), has been developed to assess aspects of memory, attention, planning and reasoning in healthy adults and patient populations (Owen et al., 1990, 1991, 1992, 1996, 2010; Bor et al., 2003; Hampshire et al., 2012). The CBS tests have been validated in patients with anatomically-specific brain lesions (e.g., Owen et al., 1990, 1991), in neurodegenerative populations (e.g., Owen et al., 1992, 1993), in pharmacological intervention studies (e.g., Lange et al., 1992; Mehta et al., 2000), and their neural correlates have been well-studied using functional neuroimaging in healthy adults (e.g., Owen et al., 1996), and in neuropathological populations (e.g., Owen et al., 1998; Williams-Gray et al., 2007). Recently, the tests were adapted to run online without formal supervision, opening the possibility of large-scale studies of cognition in the general population (Hampshire et al., 2012; Wild et al., 2018). In a recent pilot study (Honarmand et al., 2019), this cognitive battery of tests, called Cambridge Brain Sciences (CBS), was used in a cohort of ICU patients, found that it is both feasible to self-administer and able to identify cognitive impairments in several domains. The CBS tests have now been taken more than 10 million times, generating one of the largest databases of its kind in the world. Crucially, this includes a normative database of 75,000 participants. These statistics demonstrate that web-based studies of cognition are not only possible, but provide a novel opportunity for assessing the multitude of factors that contribute to healthy cognition on a scale that would be simply impossible using traditional laboratory-based methods.

By bridging the gap between novel high-density EEG markers and rigorous, long-term tracking of cognitive recovery, the current study aims to design a point-of-care system that predicts neurological outcomes of continuously-sedated, brain-injured patients in the ICU. More specifically, we aim to identify EEG markers that can be recorded at the ICU bedside, which (1) capture network reorganization upon the change of sedation (i.e., during a brief interruption of continuous sedation), and (2) robustly predict recovery of consciousness and cognition up to 12 months post-injury. To attain this objective, we will carry out high-density EEG recordings between 24 h and 7 days post-ICU admission on continuously sedated brain-injured patients before, during and after a brief interruption of continuous sedation. Patients who subsequently regain consciousness will be asked to complete an abridged CBS battery on a daily basis during the hospital stay and on a weekly basis up to 3 months following the recovery of consciousness (i.e., start of CBS testing). Testing will continue on a monthly basis until 12 months post-injury.

Findings from this study will have important implications for our understanding of how the functional EEG networks that may underlie consciousness and cognition are disrupted by injury, and how these networks drive recovery. Ultimately, this study has the potential to improve clinical decision-making and prognostication of unresponsive, brain injured-patients, within the first days post-injury.

## Materials and Equipment

This study requires a high-density EEG system comprising a minimum of 64 channels, headphones, and a tablet or laptop computer with Internet access.

### Methods

#### Ethics and Study Design

This multi-center study was reviewed and approved by the Research Ethics Board of the McGill University Health Centre (Project ID 2020-5972) and the Western University Health Science Research Ethics Board (Project ID 114303). Given that research participants are unable to consent to their participation in the study given the sudden nature of their injury or medical condition, written informed consent will be provided by a family member (next of kin) in accordance with the Declaration of Helsinki.

#### Participants

Two hundred (*n* = 200) brain-injured patients in a pharmacologically-induced coma will be recruited from three ICUs in Montreal, QC, Canada (the Montreal General Hospital, the Montreal Neurological Hospital, the Royal Victoria Hospital) and two ICUs in London, ON, Canada (University Hospital and Victoria Hospital). Sample size was determined based on feasibility (i.e., number of patients in this condition per ICU on a yearly basis, estimated patient survival rates) and the fragile medical state of our study population. Considering that several patients may not survive their injury and/or may withdraw from the study prior to the 12-month follow-up, we aim to have 200 patients initially included in order to have 100 patients complete the entire study (up to 12 months post-injury).

Patients will be included if they have suffered a brain injury (e.g., traumatic brain injury, anoxic brain injury, stroke, subarachnoid hemorrhage); are continuously sedated; are between 24 h and 7 days post-injury; are aged between 18 and 70 years of age; and have a planned interruption of sedation for clinical assessment. Patients will be excluded if: they are not native English or French speakers; they are deemed medically unsuitable by the attending physician; there are barriers to appropriate application of the high-density EEG net (e.g., scalp infections, burns); they have a history of pre-existing dementia or mild cognitive impairment; they show presence of *status epilepticus*; they are on contact precautions (e.g., methicillin-resistant *Staphylococcus aureus*, COVID-19), which require an extraordinary disinfection protocol for testing equipment.

#### Protocol

**FIGURE 1:**
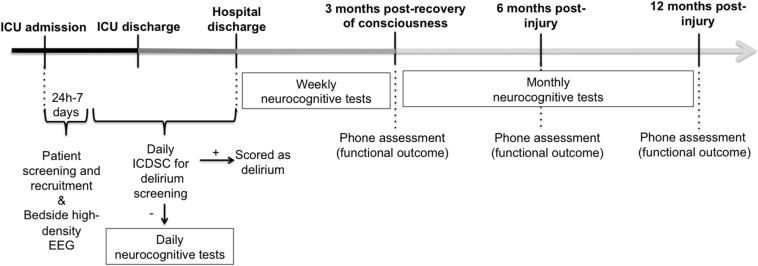
General timeline of overall study measures. Continuously-sedated brain-injured patients will be recruited from the ICU and have their brain activity recorded before, during and after a brief interruption of continuous sedation, using high-density EEG between 24 h and 7 days post-injury. Once patients recover consciousness, they will be screened daily for delirium. Once they have been confirmed delirium-free, they will begin taking neurocognitive tests using the Cambridge Brain Sciences (CBS) battery on a daily basis, or as often as possible, until there are discharged from the hospital. Up to 3 months following their first CBS test, patients will continue to take the CBS battery on a weekly basis. After the initial 3 months of intensive neurocognitive testing, patients will be asked to complete the CBS battery on a monthly basis, up to 12 months post-injury. A phone assessment will also take place 3 months following the recovery of consciousness (i.e., start of CBS test), and at 6 and 12 months post-injury, to assess functional outcome using the Glasgow Outcome Scale-Extended and the Disability Rating Scale. ICDSC, Intensive Care Delirium Screening Checklist; ICU, Intensive Care Unit.

The overall protocol timeline is detailed in Figure 1. Briefly, between 24 h and 7 days post-ICU admission, high-density EEG will be recorded from continuously-sedated brain-injured patients before, during, and after a brief interruption of their pharmacological sedative. Brief interruptions of sedation are routinely performed in standard clinical practice for most brain-injured patients in the ICU to assess for clinical stability and behavioral reactivity. Among patients who subsequently regain consciousness during or following their ICU stay, we will prospectively track neurocognitive and functional outcomes up to 12 months post-injury, using an abridged CBS battery and follow-phone interviews.

#### Demographic and Clinical Characteristics

For screening and to characterize the association between our EEG and neurocognitive markers and demographic and acute critical illness characteristics, we will record: age; sex; occupation; level of education; ethnicity; languages spoken; pre-morbid medical history (including neurocognitive and psychiatric disorders); and pre-morbid substance use; injury type (e.g., traumatic, anoxic); mechanism of injury; Glasgow Coma Scale score; severity illness score (SOFA and MODS); CT scan findings (e.g., Marshall and Rotterdam scores); presence and duration of intracranial pressure (if monitored); visual fixation; sedation/analgesia drug levels; delirium duration; post-traumatic amnesia duration; ICU stay duration, and hospital stay duration. For all sedatives and analgesics received in the 24 h prior to the EEG recording, we will also record the doses, types and administration routes and times.

#### High-Density EEG

**FIGURE 2:**
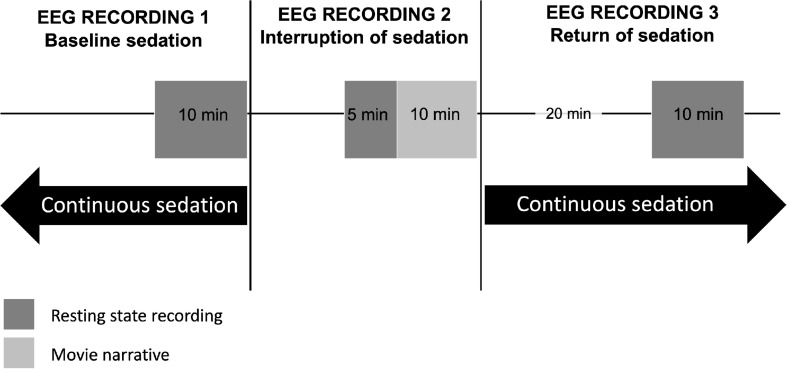
Schematic timeline of EEG recordings before, during, and after a brief interruption of continuous sedation. In the ICU, between 24 h and 7 days post-injury, continuously-sedated brain-injured patients will have their brain activity recorded using high-density EEG during 10 min at resting state, under continuous sedation (EEG Recording 1). The clinical team will then interrupt sedation to carry out their neurobehavioral assessment, as standard of care. Following this assessment, sedation will continue to be withheld for 15 additional minutes, during which EEG will be recorded for 5 min at resting state, and 10 min during which an auditory narrative will be played through headphones, in its scrambled (5 min) and intact form (5 min) (EEG Recording 2). Twenty minutes after continuous sedation is reinstated, a final 10-min resting state EEG recording will take place (EEG Recording 3).

This protocol will be conducted using a 128-channel EEG recording system from Electrical Geodesics, Inc. (Eugene, OR, United States) with sponge-based electrode nets, which provides the ability to set up all electrodes in approximately 10 min. Consented participants at each site will be outfitted with this EEG system at their bedside 30 min prior to a planned clinical cessation of continuous sedation. The bedside EEG recording will take place in three phases: (1) baseline continuous sedation (i.e., when patients are continuously sedated); (2) interruption of sedation (i.e., after continuous sedation has been interrupted for clinical assessment); and (3) return of sedation (i.e., 20 min after continuous sedation has been restarted) (see Figure 2).

##### EEG recording 1 – baseline continuous sedation

Prior to the scheduled cessation of sedation, participants will have their brain activity recorded for 10 min at resting state.

##### EEG recording 2 – interruption of sedation

Sedation will be stopped, and clinicians will conduct their clinical neurobehavioral assessment, as per standard of care. The EEG net will remain on the participant’s head during this time. Once sedatives have been withheld and the clinical neurobehavioral assessment has been completed, participants will remain without sedation for a maximum of 15 additional minutes – or as long as the patient is tolerating it and the medical team deems it safe [i.e., vitals remain stable and intracranial pressure remains normal (<20 mmHG)] – during which brain activity will be recorded with EEG for the study. Of these 15 min of EEG recording, 5 min will be recorded at resting state and 10 min will be recorded while the patient is listening to an auditory narrative intended to evoke evidence of higher cognitive functioning, including covert awareness (Naci et al., 2017). We will use the auditory narrative paradigm to look for evidence of covert awareness during the interruption of continuous sedation. Patients will have headphones placed over their ears for 10 min while EEG is recorded during the interruption of sedation. We will play a 5-min scrambled audio segment of the motion picture *Taken*, and subsequently play the unscrambled audio of the same 5-min segment.

As patient safety will be prioritized, sedatives will be reinstated as standard of care after the 15 min of EEG recording, or as soon as the clinical team decides to do so, whether or not the EEG recording is complete.

##### EEG recording 3 – return of continuous sedation

Twenty minutes following the return of continuous sedation, participants will have their brain activity recorded for 10 min at resting state, at which point the EEG recording will be discontinued.

### EEG Data Analysis

#### EEG Pre-processing

Data will be pre-processed using EEGlab (Delorme and Makeig, 2004). Data will be bandpass filtered between 0.1 and 50 Hz and all artifacts will be removed. All non-brain electrodes will be removed from subsequent analyses. Data will be segmented into the three phases of the EEG recording: (1) baseline continuous sedation; (2) interruption of sedation; and (3) return of continuous sedation.

#### Spectrogram and Topographic Maps

Spectrograms ranging from 0.1 to 50 Hz, and topographic maps of each frequency band [delta (1–4 Hz), theta (4–8 Hz), alpha (8–13 Hz), beta (13–30 Hz)], and phase-amplitude coupling measures will be used to characterize the EEG in each phase, and will be calculated semi-automatically through the designated pipeline EEGapp (Biosignal Interaction and Personhood Technology Lab, McGill University).

#### Functional Connectivity

Functional connectivity between all potential electrode pairs will be assessed with the weighted phase lag index (wPLI) (Vinck et al., 2011), which avoids confounds of volume conduction (Stam et al., 2007). First, cleaned EEG data will be segmented into delta (1–4 Hz), theta (4–8 Hz), alpha (8–14 Hz), and beta (14–30 Hz) frequency bands using band-pass filtering methods. wPLI will be calculated using the following formula:

$$w\,P\,L\,I_{i\,j}=\frac{\left|E\,\left\{𝒥\,\left(C_{i\,j}\right)\right\}\right|}{E\,\left\{\left|𝒥\,\left(C_{i\,j}\right)\right|\right\}}=\frac{\left|E\,\left\{\left|𝒥\,\left(C_{i\,j}\right)\right|\,s\,g\,n\,\left(𝒥\,\left(C_{i\,j}\right)\right)\right\}\right|}{E\,\left\{\left|𝒥\,\left(C_{i\,j}\right)\right|\right\}}$$

where *𝒥*(*C*_*ij*_) is the imaginary part of cross-spectrum *C*_*ij*_ between signals *i* and *j* (Vinck et al., 2011). The cross-spectrum *C*_*ij*_ is defined as Z*_*i*_*Z*_*j*_*^∗^, where Z*_*i*_* is the complex value Fourier spectra of the signal i for each frequency, and Z*_*j*_*^∗^ is the complex conjugate of Z*_*j*_*. *C*_*ij*_ can be written as *Reiθ*, where R is magnitude and *θ* is the relative phase between signal *i* and *j* (Vinck et al., 2011). A wPLI value of 1 indicates complete phase locking between the two signals (i.e., that the instantaneous phase of one signal is leading the other). Conversely, a wPLI value of 0 indicates no consistent phase-lead or -lag relationship.

In order to characterize the direction of functional connectivity (i.e., the direction of phase-lead/lag relationship between channels *i* and *j* in the wPLI matrix), we will calculate the directed phase lag index (dPLI) (Stam and van Straaten, 2012). The instantaneous phase of each EEG channel will be extracted using a Hilbert transform, and the phase difference Δ*φ*_*t*_ between all the channels will be calculated where Δφ_*t*_ = φ_*i*,*t*_−Δφ_*j*,*t*_, t = 1,2,…,N, where N is the number of samples in one epoch, and *i* and *j* include all channels. dPLI will then be calculated using the following formula:

$$d\,P\,L\,I_{i\,j}=<H\,\left(\Delta \,\varphi_{t}\right)>$$

where H(x) represents the Heaviside step function, where H(x) = 1 if x > 0, H(x) = 0.5 if x = 0 and H(x) = 0 otherwise. A dPLI value ranging from 0.5 to 1 will indicate that signal *i* leads signals *j*, whereas a dPLI value between 0 and 0.5 will indicate the reverse. If there is no phase-lead/phase-lag relationship between signals, dPLI = 0.5.

For both the wPLI and dPLI matrices, we will control for noise-induced phase relationships using surrogate datasets in which the phase relationship between two channels will be randomized, but their spectral properties maintained. Data segments used to generate surrogate wPLI/dPLI matrices will be 10 s in length, and will be permuted 20 times to generate a distribution of values representing the spurious connectivity. The wPLI and dPLI values of the original, non-shuffled EEG data will then be compared to this distribution of surrogate data using a Wilcoxon signed rank test and will be set to 0 (wPLI) or 0.5 (dPLI) if they do not achieve statistical significance.

#### Graph Theoretical Brain Network Properties

The functional brain network of each frequency band (i.e., delta, theta, alpha, beta) will be constructed using the wPLI of all pairwise combinations of electrode channels. We will then construct a binary adjacency matrix A_*ij*_ using a custom threshold for each participant: if the wPLI*_*ij*_* value of nodes *i* and *j* are above the custom threshold of all wPLI values, A*_*ij*_* = 1; otherwise, A*_*ij*_* = 0. The custom threshold will be determined by identifying the lowest threshold enabling a minimally-spanning graph during the interruption of sedation, which will be considered the more naturalistic brain network of the three recordings. This will enable a more accurate construction of each patient’s brain network, based on how their brain injury affects connectivity characteristics. From the binary adjacency matrix, we will calculate basic graph theoretical network properties, including global efficiency (Latora and Marchiori, 2001), clustering coefficient (Watts and Strogatz, 1998), modularity (Newman, 2006), and binary small-worldness (Humphries and Gurney, 2008).

Hub nodes are highly-connected nodes, considered to play a vital role in the structure of a network and the flow and integration of information within the network (van den Heuvel and Sporns, 2013). In healthy individuals, their structure and topographical location become altered under anesthesia (Lee H. et al., 2013) Using the network of each frequency band, network hubs will be calculated using node degree (i.e., the number of edges connected to a node) and betweenness-centrality (i.e., the number of shortest paths passing through a node).

### EEG Features Across Changes in Sedation Status

Changes in the aforementioned EEG features (i.e., spectrograms, topographic maps, wPLI, dPLI, graph theoretical network properties and hubs) between the three phases (i.e., baseline continuous sedation; interruption of sedation; and return of continuous sedation) will be used to assess the brain’s ability to adaptively reconfigure its networks in response to changes in level of anesthesia; the degree of adaptive reconfiguration will be used as predictors of outcome.

### Auditory Narrative – Inter-Subject Correlations and Correlated Components Analysis

We will examine the inter-subject synchronization between each patient and healthy participants (database already acquired). The data will be interrogated using a ‘bottom up’ data-driven correlated components analysis (CorrCA; Dmochowski et al., 2012; Cohen and Parra, 2016; Ki et al., 2016), which is ideal for continuous naturalistic stimuli like movie narratives (Laforge et al., in press). The CorrCA is one method to calculate inter-subject neural synchronization (inter-subject correlations)—a common index of shared neural processing during movie tasks—across a group (Dmochowski et al., 2012; Naci et al., 2014, 2017; Ki et al., 2016). The basic CorrCA procedure is as follows: the CorrCA is a non-parametric data reduction technique that extracts a pattern of electrode activity that is maximally correlated across participants during the movie task. This pattern represents the common neural response to its auditory and linguistic features and importantly, its plot (Dmochowski et al., 2012; Cohen and Parra, 2016; Ki et al., 2016). Like other component extraction methods (e.g., PCA, ICA), the CorrCA does this through an eigenvalue decomposition of covariance data, only here, it uses the pooled within-subjects cross covariance

$$R_{w}=\frac{1}{N}\,\sum_{k=1}^{N}R_{k\,k},$$

and between-subjects cross covariance

$$R_{b}=\frac{1}{N\,\left(N-1\right)}\,\sum_{k=1}^{N}\sum_{l=1,l\neq k}^{N}R_{k\,l}$$

where

$$R_{k\,l}=\sum_{t}\left(x_{k}\left(t\right)-{\bar{x}}_{k}\right)(x_{l}\left(t\right)-{\bar{x}}_{l}^{T}$$

to calculate the cross-covariance across all electrodes x and time *t* between participant *k* and *l*. Therefore, eigenvectors w*_*i*_* of the cross-covariance matrix R*_*w*_*^–1^R*_*b*_*, with the largest eigenvalues λ*_*i*_* computed as (R*_*w*_*^–1^R*_*b*_*)w*_*i*_* = λ*_*i*_*w*_*i*_* are the components that maximize Pearson’s rho between subjects (Dmochowski et al., 2012; Cohen and Parra, 2016; Ki et al., 2016). The CorrCA produces *N-1* components that are rank-ordered by the magnitude of their correlation across subjects, where *N* is the number of input sources (electrodes). The spatial weights of the top-ranked group-level component can then be back-projected onto the EEG data from individual subjects—effectively creating a spatial filter of the data—to generate a component time course for each participant. We will then correlate the individual component time courses across the group and compute the average correlation coefficient on a per-subject basis to compute individual inter-subject correlations which reflects the similarity of each participant’s neural activity relative to the group (Cohen and Parra, 2016; Ki et al., 2016; Iotzov et al., 2017).

The reliability of the CorrCA component and the statistical significance of the inter-subject correlations can be established using a leave-one-out variant of the CorrCA and permutation statistics. Specifically, the CorrCA is iteratively calculated on all possible *N-1* subsets of the group. This produces unique components and time courses for each subset which can be used to calculate the average inter-subject correlations for each participant *across* subsets. Statistical significance of the per-subject means inter-subject correlations can then be determined by comparing their magnitude to the top 5% (*p* < 0.05, FDR corrected) of a null distribution of phase-shifted correlation coefficients generated using a 1000 iteration resampling procedure (Theiler et al., 1992). This analysis technique will be applied to the EEG data from individual patients to compare their neural activity during the intact and scrambled versions of *Taken*.

### Neurocognitive and Functional Outcomes

**FIGURE 3:**
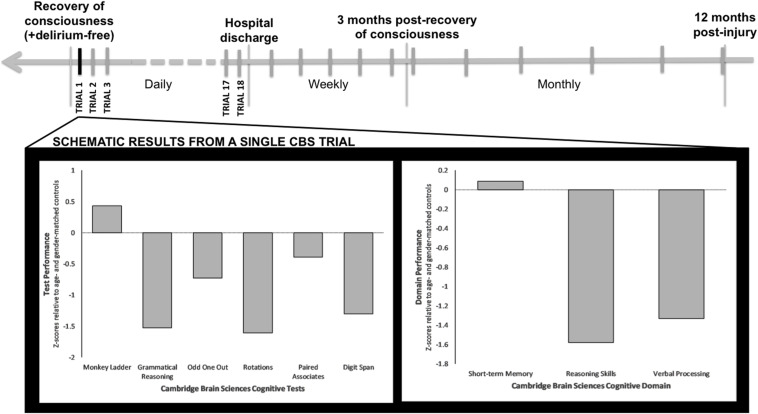
Schematic timeline of CBS testing and results per trial. Once patients recover consciousness, are delirium free and able to carry out the CBS web-based battery, testing will take place on a daily basis until hospital discharge. After hospital discharge and until 3 months following Trial 1 of CBS testing, testing will take place on a weekly basis. After the first 3 months of testing, CBS tests will then be taken on a monthly basis, up to 12 months following injury. Displayed is a single CBS trial for an ICU patient with a primary brain injury who has recovered consciousness. This patient’s test-specific (lower left) and domain-specific (lower right) results are plotted in relation to normative data for the patient’s age and sex.

We will prospectively track neurocognitive outcomes up to 12 months post-injury, using the web-based CBS platform (Cambridge Brain Sciences, 2020) (see Figure 3). This will enable patients to complete the tests from any location (e.g., ward, rehabilitation facility, home) without the need to attend clinic, thereby overcoming current limitations of standard cognitive batteries and enabling inclusion of previously inaccessible ICU survivors.

Once patients are recruited in the ICU and have recovered consciousness, they will be screened daily for delirium using the Intensive Care Delirium Screening Checklist (ICDSC) (Bergeron et al., 2001). If they score negative, they will be asked to provide written informed consent to continue participation in the study, or to withdraw. If they choose to continue participating in the study, they will be asked to complete an abridged version of the CBS battery on a laptop or tablet (as soon as able). This battery of six tests will take up to 20 min to complete. The tests include: Odd One Out, Rotations, Paired Associates, Grammatical Reasoning, Monkey Ladder, and Digit Span (see Wild et al., 2018). These tasks are designed to assess verbal and deductive reasoning, episodic memory, visuospatial working memory, and short-term memory (see Table 1). CBS testing will be repeated every day during the acute phase (ICU, ward), or as often as possible given the constraints of ICU and ward care. Following hospital discharge, patients will receive a URL link via email to complete the same test series online on a weekly basis for up to three months following their first CBS trial. After this period, patients will be asked to complete neurocognitive tests every month for up to 12 months post-injury.

**TABLE 1:** Tests comprising the abridged version of the Cambridge Brain Sciences battery used in this study.

Name	Descriptions
Odd one out	Based on a sub-set of problems from the Cattell Culture Fair Intelligence Test (Cattell, 1949). Nine groups of colored shapes are displayed in a grid. The features define each group (color, shape, # of items) are related to each other according to a set of rules. Participants must deduce the rules that relate these features and select the group whose contents do not correspond to those rules. They have 90 s to solve as many problems as possible, and the puzzles get progressively more difficult. A correct response increases the final score by one point, whereas an incorrect response decreases the score by one point.
Rotations	Task that measures the ability to spatially manipulate objects in mind (Silverman et al., 2000). On each trial, two groups of colored squares (each with N squares) are displayed beside each other. One of the groups is rotated by a multiple of 90 degrees. The groups are either identical (when un-rotated) or differ by the position of just one item, and participants must indicate if the groups match. They have 90 s to complete as many trials as possible. A correct response increases the final score by N, and the subsequent trial has groups of N+1 squares. If the response is incorrect, the total score decreases by N, and next trial has groups of N-1 squares.
Paired associates	Based on a test commonly used to assess memory impairments in aging clinical populations (Gould et al., 2005). Sets of boxes are displayed at random locations on grid. The boxes open one after another to reveal an icon, after which they close. The icons are then displayed sequentially in the center of the screen, and the participant must select box that contained that icon. If the participant remembers all the icon-location pairs correctly, then the next trial will have one more box. If an error is made the next trial has one less box. The test ends after three errors. The participant’s score is the maximum number of pairs successfully remembered.
Grammatical reasoning	Based on Alan Baddeley’s 3-min grammatical reasoning test (Baddeley, 1968). On each trial, a written statement regarding two shapes is displayed on the screen, and the participant must indicate whether it correctly describes the shapes pictured below. The participant has 90 s to complete as many trials as possible. A correct response increases the total score by one point, and an incorrect response decrease the score by one point.
Monkey ladder	Based on a task from the non-human primate literature (Inoue and Matsuzawa, 2007). Numbered boxes are displayed (at the same time) at random locations within a grid. After a variable interval (number of squares * 900 ms), the numbers disappear leaving only the boxes. Participants must click the boxes in ascending numerical sequence. Difficulty is varied dynamically like in Spatial Span. The test finishes after three errors, and the resulting score is the length of the longest sequence successfully remembered.
Digit span	Based on the verbal working memory component of the WAIS-R intelligence test (Weschler, 1981). A sequence of digits is displayed, one at a time, in green in the center of the screen. Participants must then repeat the sequence of digits by selecting them on the on-screen keyboard. Difficulty is dynamically varied like previous tests, and the test ends after three mistakes. The resulting score is the length of the longest digit sequence successfully remembered.

Functional outcomes will also be assessed through brief phone interviews at 3 months post-recovery of consciousness (i.e., start of CBS testing), and at 6 and 12 months post-injury. The scales used for this assessment will be the Disability Rating Scale and Glasgow Outcome Scale-Extended (Rappaport et al., 1982; Wilson et al., 1998). Pre-injury occupation will be compared to occupation 6 and 12 months post-injury, to assess functional recovery compared to pre-injury status.

### Association Between Acute EEG Features and Patient Outcomes

A machine learning approach will be used to determine which EEG features are most predictive of patient outcomes and long-term cognitive recovery. We will investigate various types of classification algorithms that can be applied to both bi-class (recovery of consciousness vs. non-recovery of consciousness; survival vs. death) and multi-class classification, based on different levels of cognitive and functional recovery. These algorithms will include generative and discriminative modeling approaches, such as linear discriminant analysis, support vector machines and decision trees. To account for the high degree of correlation between trials collected from a single participant, the performance of the proposed classification methods will be evaluated using a leave-one-subject-out cross-validation procedure, when applicable.

Performance accuracy will be assessed by testing if the degree of acute EEG reconfiguration and the level of plot following upon interruption of sedation can accurately predict outcome and cognitive recovery at ICU and hospital discharge, and at 3, 6, and 12 months post-recovery of consciousness. For each of the classifiers used on our dataset, we will also generate bootstrap confidence intervals. The bootstrap dataset will be created by sampling with replacement from the original dataset until a new dataset (containing duplicate samples) of the same size is generated. The bootstrap dataset will be separated into training and test sets, and used to characterize the performance of each machine learning algorithm tested. This process will be repeated 5000 times, creating a distribution of the classifier performance. Lower and upper bounds for the bootstrap confidence interval will be set at the 2.5^th^ and 97.5^th^ percentile, corresponding to *p* ≤ 0.05. Two classifiers will be considered to have statistically different levels of performance if their confidence interval does not overlap with a bootstrap resampling of 5000. The classifier with the highest performance accuracy will be used to generate the final results describing the association between acute EEG features and patient outcomes.

### Association Between Demographic/Clinical Characteristics and Patients’ EEG Features and Outcomes

In order to investigate the effect of clinical and demographic characteristics on EEG reconfiguration during interruption of sedation and on cognitive recovery trajectories, linear regression will be performed separately for each of these variables, with models constructed as follows: the variables sex (male/female), bilingualism (monolingual/multilingual), presence of pre-morbid neurocognitive or psychiatric disorder (presence/absence), presence of visual fixation at 24 h post-injury (yes/no), presence of delirium (yes/no), and presence of elevated intracranial pressure (yes/no) will be used as binary regressors. The variables age (mean-centered across entire sample), education, ethnicity, injury type, GCS at admission, severity illness score, Marshall and Rotterdam scores, duration of elevated intracranial pressure, delirium duration post-traumatic amnesia duration, ICU stay duration, and hospital stay duration will be treated as categorical, with n-1 regressors. All statistical tests will be corrected for multiple comparisons using a False Discovery Rate across scores, and separately for each effect. We include measures of standardized and unstandardized effect sizes, confidence intervals, and Bayes factors.

### Anticipated Results

**FIGURE 4:**
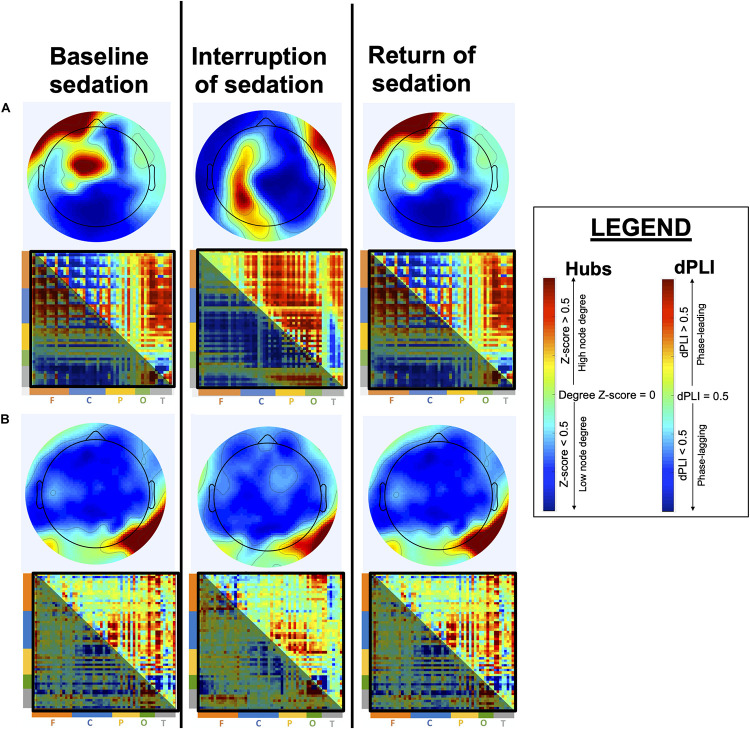
Anticipated brain network reconfiguration across states of sedation. This figure depicts expected network reconfiguration across sedation states, in alpha network hubs and directed Phase Lag Index (dPLI). Topographic hub maps show node degree, with highest-degree nodes in red and lowest-degree nodes in blue. dPLI matrices depict the direction of functional connectivity between two nodes, organized by region (F = frontal; C = central; P = parietal; O = occipital; T = temporal). Red indicates phase-leading connectivity (row-to-column) whereas blue indicates phase-lagging connectivity (row-to-column), such that a red-orange upper part of the matrix (above the diagonal) indicates feedback-dominant connectivity. Specifically, in patients who will eventual recover optimally, we expect alpha network hubs to shift from an anterior position during baseline sedation to a posterior location during interruption of sedation, and to return to their baseline position upon the reinstatement of continuous sedation **(A)**. Moreover, we expect dPLI to be mainly feedforward-dominant during baseline continuous sedation and to show an increase in feedback-dominance connectivity during interruption of sedation. In patients who will not recover consciousness, or show poor cognitive recovery **(B)**, we expect both alpha network hubs and dPLI to show little to no reconfiguration during the interruption of sedation.

Our general hypothesis is that the brain’s ability for adaptive network reconfiguration upon interruption of continuous sedation will be associated to the eventual recovery of consciousness and to more favorable cognitive and functional outcomes. We expect that patients who recover consciousness and demonstrate the strongest cognitive recovery will have brain network and connectivity markers that re-appear during the brief interruption of continuous sedation. More specifically, we expect the re-appearance of feedback-dominant connectivity between the frontal and parietal regions and the presence of posterior alpha network hubs to be strongly associated to patient prognosis (i.e., recovery of consciousness and cognitive functions) (Figure 4).

**FIGURE 5:**
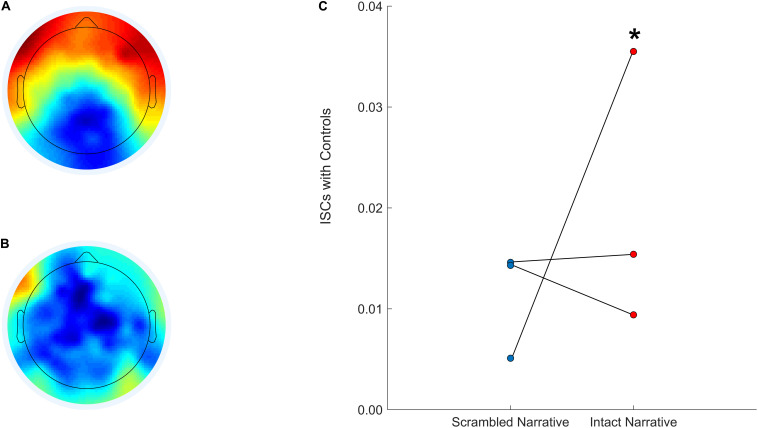
Anticipated component topographies and patient inter-subject correlations with controls during the scrambled and intact *Taken* audio. The CorrCA component topography calculated for a control group (EEG data from *N* = 15 healthy participants) during the intact version of *Taken* **(A)**. The CorrCA component topography calculated for a control group (EEG data from *N* = 15 healthy participants) during the scrambled version of *Taken* **(B)**. Three patients’ expected inter-subject correlations with controls after back-projection for the scrambled (left; blue) and intact (right; red) versions of *Taken* **(C)**. *Denotes hypothetical patient who would be expected to have more favorable cognitive and functional recovery.

We can also develop similar hypotheses regarding the auditory narrative paradigm; we predict that an increase in inter-subject correlations after sedation is interrupted will be associated with more favorable outcomes. This would reflect a functional reconfiguration of the pathways that support basic sensory processing and more complex cognitive functions like speech perception, attention, and short-term memory—all of which are necessary for narrative processing (Ki et al., 2016; Baldassano et al., 2017). This approach is expected to yield results similar to what is depicted in Figure 5. Specifically, we will back-project the normative component topographies for the intact (5A) and scrambled (5B) versions of the audio onto EEG data acquired from patients and calculate their inter-subject correlations with the control group. We expect patients with higher inter-subject correlations during the interruption of sedation (5C) to have more favorable outcomes and recovery trajectories.

Finally, by monitoring the natural history of cognitive recovery in our patients, one of our expected outcomes is to be able to determine how patient, injury and treatment characteristics impact cognitive recovery in ICU survivors. More specifically, we expect to identify demographic, injury, and treatment-related characteristics that mediate or moderate recovery trajectories and outcomes.

This study has the advantage of relying on accessible methods and equipment. Firstly, a brief interruption of continuous sedation is routinely performed in standard clinical practice for most brain-injured patients in the ICU to assess behavioral responsiveness. This study will be carried out only on patients who have a scheduled interruption of sedation by the medical team, and will therefore not incur any additional medical procedures. Second, EEG is an accessible neurophysiological assessment tool, which can easily be used at the bedside. Following this study, the EEG features identified as most predictive of patient outcome will be adapted to the lower-density (e.g., 16 channel) EEG systems that are typically deployed in the ICU, and custom software will be developed that calculates these features on a desktop computer that can be brought to the ICU bedside. The present study will therefore have immediate translational potential, even in ICUs that do not have access to a high-density EEG system. Finally, on a practical level, this study will validate a short, web-based cognitive battery that can be used immediately by ICU survivors to chart their recovery at home and at no cost. Current neurocognitive tools are expensive and inconvenient; they are time consuming, require specially trained staff to administer, and subjects must attend testing sessions in person. As a result, the subject pool for current neurocognitive tools largely excludes patients who are institutionalized, have limited mobility, those who do not live near the testing center, or who have negatively associated traumatic memories due to their recent hospital experience, and are unwilling to return to the admitting hospital (Honarmand et al., 2020). Access to web-based neurocognitive battery therefore has valuable implications for this patient population.

As this is a prognostic study on acute ICU patients, we expect that a large proportion of our patients will not survive. However, this study will monitor all patient outcomes, including death. Indeed, we expect our EEG features to be associated to all patient outcomes, from death to full cognitive recovery. We also expect to have some patients with promising EEG findings for whom there will be a decision for withdrawal of life sustaining therapies based on patient values. Based on a recent meta-analysis, average mortality rates for severe traumatic brain injury was 39% while unfavorable outcome was 60% (Rosenfeld et al., 2012). Thus we anticipate approximately 50% of our initial sample (*n* = 100) will be able to complete neurocognitive testing. In itself, this is a larger sample size than most studies that prospectively assess ICU patient populations.

## Discussion

This study is designed to develop a point-of-care system that can accurately predict outcomes of unresponsive, brain-injured patients in the ICU. This system will combine (1) advanced techniques in EEG network analysis with (2) a web-based battery of cognitive tests for long-term assessment of cognitive recovery in ICU survivors, to create a method of accurately predicting patient prognosis and long-term outcomes.

One important limitation to this study is that several patients will undergo withdrawal of life support measures and palliation that may result in death prior to completion of the study follow up. Withdrawal of care will necessarily introduce an important bias in our machine learning classification, as it will remain impossible to know if these patients could have recovered cognitively. However, these patients will be classified differently than patients who recover or die naturally, so as to avoid blurring the interpretation or classification accuracy of our models (i.e., survived to hospital discharge; death in ICU; death in hospital; withdrawal of life support measures in the ICU resulting in death prior to ICU (or hospital) discharge). Moreover, the heterogeneity of our patient population may also limit the interpretation of our results. For example, we will recruit patients in a very wide age range (18–70), and recovery speed and trajectories are expected to significantly vary with age. Conversely, injury type will also vary within our sample. However, we expect our within-subject approach to be able to address this limitation, given that participants will serve as their own baseline in terms of EEG response to the temporary interruption of sedation. Moreover, the slope of cognitive recovery trajectories will be assessed, effectively characterizing each participant’s recovery with respect to their post-injury baseline. The cognitive functions of study participants obtained through the CBS tests will be compared to age-matched normative data from healthy controls, for which 100s of 1000s of results have already been acquired. We also plan to address the heterogeneity of brain injuries by carrying out secondary analyses within subgroups of patients with the same injury type (i.e., traumatic brain injury; hypoxic ischemic brain injury; anoxic brain injury; intracerebral hemorrhage (excluding trauma)), to identify the best prognostic markers for each type of brain injury. Ultimately, although participant heterogeneity is a limitation, this study sample will be representative of continuously-sedated ICU patient populations, and thus improve the translatability of our results to clinical practice. Another study limitation is that some of our patients will have very focal injuries, while others will have diffuse injuries – the type and location of injury will undoubtedly affect recovery potential and the interpretability of our EEG results, which will not be source localized. Focal brain injuries may also lead to important hemispheric differences in our EEG results. We will address this limitation by focusing our analyses on the healthiest hemisphere, when applicable. The healthiest hemisphere will be defined according to CT scan results whenever possible, or will be based on the hemisphere showing the healthiest reorganization across the three EEG recordings of the study.

In addition to providing critically needed support for clinical decision-making, this study has the potential to transform our understanding of the brain mechanisms underlying consciousness and cognition by identifying the key networks and circuits associated with their recovery. Furthermore, this project has strong potential to address the burden of ICU-related cognitive impairment, which will continue to increase in the coming decades and constitutes a public health emergency that requires urgent, innovative solutions. This project will contribute to a nuanced understanding of the natural history of ICU-related cognitive impairment, enabling the development of effective preventative, therapeutic and rehabilitative interventions.

Ultimately, this study could improve prognostication in this most challenging group of patients. We aim for our findings to lead to an increased ability to identify patients, as soon as possible after their brain injury, who are most likely to survive, and to make accurate predictions about their long-term cognitive and functional outcome.

## Ethics Statement

This multi-center study was reviewed and approved by the Research Ethics Board of the McGill University Health Centre (Project ID 2020-5972) and the Western University Health Science Research Ethics Board (Project ID 114303). Participants will provide their written informed consent to take part in this study.

## Author Contributions

AO and SB-M conceived the study. CD, LN, GL, MB, JL, MS, TG, DD, AO, and SB-M contributed to the study design. CD, LN, GL, AF, and CM helped to prepare and test equipment critical for the preparation and conduct of the protocol. CD and GL wrote the manuscript. All authors contributed to critical review of the manuscript.

## Conflict of Interest

The cognitive tests used in this study are marketed by Cambridge Brain Sciences, of which AO is the Chief Scientific Officer. Under the terms of the existing licensing agreement, AO and his collaborators are free to use the platform at no cost for their scientific studies, and such research projects neither contribute to, nor are influenced by, the activities of the company. Consequently, there is no overlap between the current study and the activities of Cambridge Brain Sciences, nor is there any cost to the authors, funding bodies, or participants who are involved in the study. The authors declare that the research was conducted in the absence of any commercial or financial relationships that could be construed as a potential conflict of interest.
